# Surgery time interval and molecular subtype may influence Ki67 change after core needle biopsy in breast cancer patients

**DOI:** 10.1186/s12885-015-1853-1

**Published:** 2015-10-30

**Authors:** Xiaosong Chen, Siji Zhu, Xiaochun Fei, David H. Garfield, Jiayi Wu, Ou Huang, Yafen Li, Li Zhu, Jianrong He, Weiguo Chen, Xiaolong Jin, Kunwei Shen

**Affiliations:** 1 Comprehensive Breast Health Center, Ruijin Hospital, Shanghai Jiaotong University School of Medicine, 197 Ruijin Er Road, Shanghai, 20025 China; 2 Department of Pathology, Ruijin Hospital, Shanghai Jiaotong University School of Medicine, Shanghai, China; 3 University of Colorado Comprehensive Cancer Center, Aurora, CO 80045 USA

**Keywords:** Breast cancer, Core needle biopsy, Ki67 change, Molecular subtype, Surgery time interval

## Abstract

**Background:**

To investigate the accuracy of core needle biopsy (CNB) in evaluating breast cancer estrogen receptor (ER), progesterone receptor (PR), HER2, and Ki67 status and to identify factors which might be associated with Ki67 value change after CNB.

**Methods:**

A retrospective study was carried out on 276 patients with paired CNB and surgically removed samples (SRS). Clinico-pathological factors as well as the surgery time interval (STI) between CNB and surgery were analyzed to determine whether there were factors associated with Ki67 value change after CNB. Five tumor subtypes were classified as follows: Luminal A, Luminal B-HER2-, Luminal B-HER2+, Triple Negative (TN), and HER2+. Ki67 value change was calculated as SRS minus CNB.

**Results:**

Mean STI after CNB was 4.5 (1-37) days. Good agreement was achieved for ER, PR, and HER2 evaluation between CNB and SRS. However, Ki67 expression level was significantly higher in SRS compared with CNB samples: 29.1 % vs. 26.2 % (P < 0.001). Both univariate and multivariate analysis demonstrated that STI and molecular subtype were associated with a Ki67 change after CNB. Luminal A tumors experienced more Ki67 elevation than Luminal B-HER2- diseases (6.2 % vs -0.1 %, *P* = 0.014). Patients with longer STI after CNB had a higher Ki67 increase: -1.1 % within 1-2 days, 2.1 % with 3-4 days, and 5.6 % more than 4 days, respectively (*P* = 0.007). For TN and HER2+ tumors, the Ki67 change was apt to be 0 with STI ≤ 4 days, while a >7 % Ki67 increase was noticed in patients with STI ≥ 5 days.

**Conclusion:**

CNB was accurate in evaluating ER, PR, HER2, and molecular subtype status. Ki67 value significantly increased after CNB, which was associated with STI and molecular subtype. Further translational research needs to consider Ki67 changes following CNB among different breast cancer molecular subtypes.

**Electronic supplementary material:**

The online version of this article (doi:10.1186/s12885-015-1853-1) contains supplementary material, which is available to authorized users.

## Background

Core needle biopsy (CNB) is recommended for an initial breast cancer pathological diagnosis and is used to evaluate estrogen receptor (ER), progesterone receptor (PR), and HER2 status [1]. Microarray data have identified that breast cancer is comprised of at least five molecular subtypes: Luminal A, Luminal B, triple negative (TN), HER2 positive and normal-like [2]. The 2013 St. Gallen breast cancer consensus recommends using ER, PR, HER2, and Ki67 results to classify breast cancer into molecular subtypes in order to guide systemic treatment decision making [3]. Our previous study showed that CNB had a high concordance rate in evaluating molecular subtype status compared with those in surgically removal samples (SRS) [4].

With the development of new agents, “window of opportunity” pre-surgical trials have been applied to test their potential anticancer abilities and mechanisms in breast cancer patients [5]. Patients in these trials are usually treated with experimental agents for a relatively short period compared with standard neoadjuvant systemic therapy [6]. In this situation, response rate is no longer suitable as an endpoint, so a breast cancer proliferation biomarker, such as Ki67, is then applied to determine the new agent’s biologic effect [7]. Thus, a Ki67 change after two weeks of endocrine treatment, for example, may predict response rates in a neoadjuvant study [8]. In addition, a decrease of Ki67 after neoadjuvant chemotherapy or endocrine therapy seems also to be related to a good prognosis [9, 10].

However, tumor heterogeneity, sample fixation, and CNB methods can cause discordance of biomarkers evaluation between CNB and SRS [4]. Also, Ki67, compared with ER, PR, and HER2, is reported to have only a fair to moderate agreement between CNB and SRS, especially in ER+/Luminal breast cancers [11, 12]. Furthermore, several studies have demonstrated that Ki67 expression will increase after CNB, which may be caused by biopsy stimulation, arguing that this Ki67 change needs to be considered in clinical practice as well as in “window of opportunity” trials [11, 13]. However, there are limited data about which factors are associated with Ki67 change after CNB. Therefore, we performed a comprehensive analysis to find which factor(s) can influence Ki67 change after CNB in early breast cancer patients.

## Methods

### Patient population

Consecutive breast cancer patients who received CNB and followed by surgery in Ruijin Hospital, Shanghai Jiaotong University School of Medicine between Oct. 2009 and Feb. 2012 were retrospectively analyzed. All enrolled patients needed paired CNB and SRS samples. Patients with large tumor were likely to receive CNB by surgeon’s choice. Ultrasound was applied to guide the CNB procedure, with more than three 14-gauce CNB samples being collected for pathological examination. CNB and SRS samples were fixed in 10 % neutral buffered formalin within 30 min after tumor removal, and fixation intervals ranged from at least 6 h to 24 h for CNB and at least 6 h to 48 h for SRS samples. Patients’ enrollment criteria were described in our previous report [11]. In addition, CNB and surgery dates were retrieved to calculate the surgery time interval (STI) after CNB. Twenty-two patients with STI more than 60 days were further excluded. All participants gave written informed consent before inclusion. The independent Ethical Committee/Institutional Review Board of Ruijin Hospital, Shanghai Jiaotong University School of Medicine reviewed and approved this study protocol, which was conducted in accordance with the Declaration of Helsinki.

### Breast cancer molecular subtype classification

The methods and positivity criteria for immunohistochemical (IHC) assessment of ER, PR, HER2, and Ki67 were described in our previous report, all of which were performed in the Department of Pathology, Ruijin Hospital, Shanghai Jiaotong University School of Medicine [11]. In brief, Ventana Autostain System (BenchMark XT, Ventana Medical Systems, Inc., Tucson, AZ) was used to stain the paired CNB and SRS, which were further evaluated by two senior pathologists (X. Fei, and X. Jin). Tumors with more than 1 % positive invasive cell nuclear staining were classified as ER+ or PR+. The 2007 ASCO/CAP (American Society of Clinical Oncology/College of American Pathologists) guidelines were applied in the HER2 status evaluation. Either HER2 IHC 3+ or fluorescence *in situ* hybridization positivity was regarded as HER2 positive (HER2+) [14]. For Ki67 expression scoring, we used the same method for calculating CNB and SRS samples. Cell distribution over the entire slice was first reviewed and 500-2000 cells were chosen from different microscopic views if the Ki67 expression distribution was uniform. Otherwise, 2000 cells were equally counted in both hotspot and negative areas in slice. Ki67 expression was scored as the percentage of positive invasive tumor cells with any nuclear staining and recorded as mean percentage of positive cells [11]. Histo-pathological parameters and receptor status in CNB were set as the baseline. Ki67 change between CNB and SRS was calculated by using CNB as the baseline.

Hormonal receptor negativity (HR-) was defined as both ER- and PR-. The concordance rate for molecular subtype classification between CNB and SRS was similar by using a Ki67 value of either 14 % or 20 %, while the latter had the higher κ value [11]. Also, 20 % was the mean value for HR+/HER2- patients and the median value for all patients in CNB samples. Thus, 20 % was selected as the Ki67 cutoff value in determining Luminal status. Five breast cancer molecular subtypes were classified according to the 2013 St. Gallen breast cancer consensus [3]: Luminal A (ER+/HER2–, Ki67 < 20 % and PR ≥20 %), Luminal B-HER2- (ER+/HER2-, Ki67 ≥ 20 % or ER+/HER2-, PR < 20 %, or ER-/PR+/HER2-), Luminal B-HER2+ (HR+/HER2+), TN (HR-/HER2–) and HER2+ (HR-/HER2+).

### Statistical analysis

Kappa test was applied to test concordance rates for ER, PR, HER2, and molecular subtypes between CNB and SRS. Values of κ > 0.6 were correlated with good agreement, values between 0.4 and 0.6 considered moderate agreements, values < 0.4 corresponded to fair, and values < 0.2 reflected poor agreement. Ki67 change after CNB was compared by using two paired samples *t* test. Chi-square test was used to calculate the association between STI and tumor characteristics. ANOVA analysis was performed to calculate the relationship between Ki67 change and potential influencing factors including: age, menopausal status, surgery type, histopathology, tumor grade, tumor size, lymph node status, ER, PR, HER2, molecular subtype, and STI. Multivariate ANOVA analysis was then done to find the association and interaction between Ki67 change and these factors. The SPSS statistical software package (version 13.0; SPSS Company, Chicago, IL) was used in the statistical analysis and two-sided *P* values less than 0.05 regarded as statistically significant.

## Results

### Patient characteristics

**Fig. 1:**
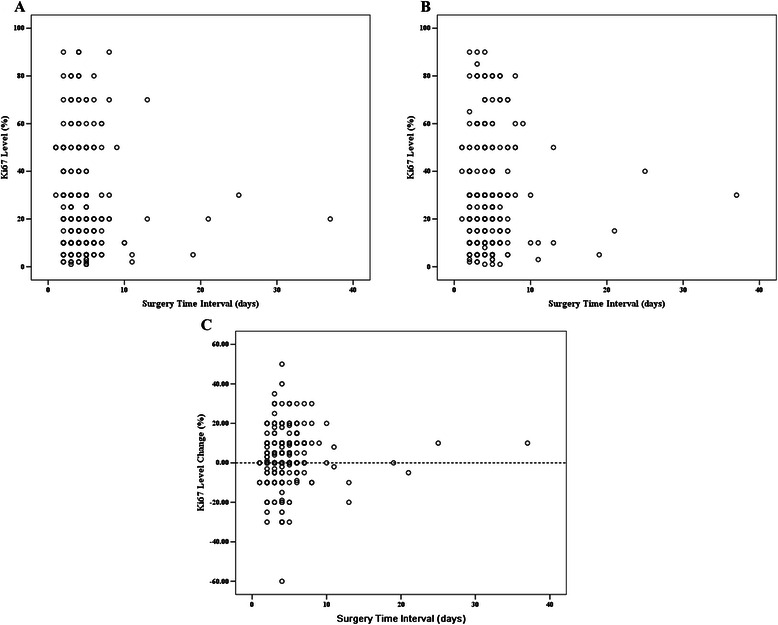
Ki67 expression level and change distribution at different surgery time intervals. **a**): Ki67 expression level of core needle biopsies versus surgery time intervals; **b**): Ki67 expression level at surgically removed samples versus surgery time intervals; **c**): Ki67 change distribution versus surgery time intervals

A total of 276 breast cancer patients were enrolled. Mean age was 56.6 (24-91) years. Ninety percent of patients were diagnosed with invasive ductal carcinoma and 32.6 % had grade III tumors. There were 214 (77.5 %) and 163 (59.1 %) of cases with ER and PR positive disease. Fifty-nine (21.4 %) had HER2+ breast cancer. Mean Ki67 value was 26.2 % (1-90 %) in CNB samples, and 53.3 % tumors were classified as Ki67 high expression. There were 73 (26.4 %), 109 (39.5 %), 33 (12.0 %), 35 (12.7 %), and 26 (9.4 %) patients classified as Luminal A, Luminal B-HER2-, Luminal B-HER2+, TN, and HER2+ subtype, respectively. Mean STI after CNB was 4.5 (1-37) days. Ten patients had STI of more than 10 days. The first, second, and third quartile days of STI were 3, 4, and 5 days, respectively. Next, we categorized STI as following groups: less than 3 days (55 patients), 3-4 days (113 patients), more than 4 days (108 patients) (Table 1). Table 2 shows STI categorized versus initial tumor characteristics as well as patient characteristics. There was no association between STI groups and clinic-pathological characteristics. Regarding Ki67 expression level at CNB and at surgery versus patients’ time to surgery, there was no significant correlation between Ki67 expression level and STI (Fig. 1a and b).

**Table 1:** Baseline patient characteristics

Characteristic	No.	Percent
Age, years	56.6 (24-91)	
<40	20	7.2
40-49	64	23.2
50-70	155	56.2
>70	37	13.4
Menstrual status		
Peri/pre-menopause	89	32.2
Post-menopause	187	67.8
Breast surgery type		
Mastectomy (+/−reconstruction)	239	86.6
Lumpectomy	37	13.4
Pathological type		
Invasive ductal carcinoma	246	89.1
Invasive lobular carcinoma	12	4.3
Others	18	6.5
Tumor size		
Tx	5	1.8
≤2 cm	118	42.8
2-5 cm	147	53.3
>5 cm	6	2.2
Axillary lymph node		
Negative	155	56.2
Positive	121	43.8
Histologic grading		
I	6	2.2
II	134	48.6
III	90	32.6
NA	46	16.7
Estrogen Receptor		
Negative	62	22.5
Positive	214	77.5
Progesterone Receptor		
Negative	113	40.9
Positive	163	59.1
Hormonal Receptor		
Negative	61	22.1
Positive	215	77.9
HER2		
Negative	217	78.6
Positive	59	21.4
Ki67 (%, mean)	26.2 (1-90)	
<20	129	46.7
≥20	147	53.3
Molecular subtype		
Luminal A	73	26.4
Luminal B-HER2-	109	39.5
Luminal B-HER2+	33	12.0
Triple negative	35	12.7
HER2 positive	26	9.4
Surgery time interval (days)	4.5 (1-37)	
1-2	55	19.9
3-4	113	40.9
≥5	108	39.1

Abbreviation: *NA* not available

**Table 2:** Surgery time interval and tumor characteristics

Characteristic	1-2 days	3-4 days	≥5 days	*P* value
Age				0.067
<40	5	5	10	
40-49	16	24	24	
50-70	32	70	53	
>70	2	14	21	
Menstrual status				0.084
Peri/pre-menopause	24	30	35	
Post-menopause	31	83	73	
Breast surgery type				0.960
Mastectomy (+/−reconstruction)	47	98	94	
Lumpectomy	8	15	14	
Pathological type				0.973
Invasive ductal carcinoma	49	101	96	
Invasive lobular carcinoma	3	5	4	
Others	3	7	8	
Tumor size				0.110^a^
Tx	2	2	1	
≤2 cm	30	45	43	
2-5 cm	23	65	49	
>5 cm	0	1	5	
Axillary lymph node				0.582
Negative	28	67	60	
Positive	27	46	48	
Histologic grading				0.821^a^
I	0	3	3	
II	30	51	53	
III	15	41	34	
NA	10	18	18	
Estrogen Receptor				0.732
Negative	11	28	23	
Positive	44	85	85	
Progesterone Receptor				0.357
Negative	21	52	40	
Positive	34	61	68	
Hormonal Receptor				0.822
Negative	11	27	23	
Positive	44	86	85	
HER2				0.797
Negative	43	91	83	
Positive	12	22	25	
Ki67 (%, mean)				0.222
<20	20	55	54	
≥20	35	58	54	
Molecular subtype				0.738
Luminal A	13	30	30	
Luminal B-HER2-	26	43	40	
Luminal B-HER2+	5	13	15	
Triple negative	4	18	13	
HER2 positive	7	9	10	

Abbreviation: *NA* not available

^a^Calculated by Fisher’s exact test

### Comparison of receptor status and Ki67 results between CNB samples and SRS

Concordance rates of ER, PR, and HER2 between CNB and SRS were 94.2 %, 87.0 % and 97.1 %. Kappa test showed κ values were 0.841, 0.729, and 0.914, respectively, demonstrating good overall agreement. Additionally, good agreement was observed for HR test, with a concordance rate of 94.2 % (κ = 0.837). In terms of molecular subtype analysis, the overall concordance rate was 72.5 %, with κ value of 0.630, also regarded as a good agreement.

Ki67 expression value was much higher in SRS compared with CNB samples by using two paired samples *t* test, with mean values of 29.1 % and 26.2 %, respectively (P < 0.001). Using 20 % as the cutoff value for a high level of Ki67 expression, the concordance rate was 80.4 %, with κ value of 0.60. TN breast cancer had the highest Ki67 value of all subtypes. Median and mean Ki67 change was 0 (inter-quartile range (IQR), -4.5 %, 10 %) and 2.9 % (±13.2 %), respectively.

### Factors associated with Ki67 change analysis

**Fig. 2:**
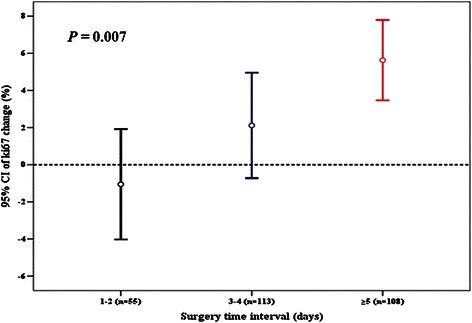
Surgery time interval and Ki67 change after core needle biopsy

**Fig. 3:**
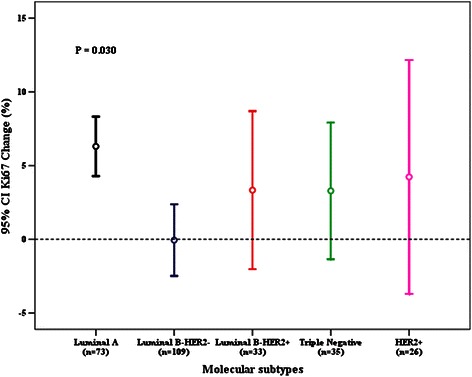
Molecular subtypes and Ki67 change after core needle biopsy

Univariate ANOVA analysis was used to determine whether patient characteristics and STI were associated with Ki67 changes. Both breast cancer molecular subtype and STI were significantly associated with Ki67 change after CNB, while other host and tumor characteristics had no influence (Figs. 2, 3, Table 3). Fig. 1c shows Ki67 change after CNB versus patients’ STI. Most cases had Ki67 change between -20 % and 20 % after CNB. Mean Ki67 change with different STIs after CNB was: -1.1 % (1-2 days STI), 2.1 % (3-4 days STI), 5.6 % (≥5 days STI), respectively (*P* = 0.007, Table 4). Subgroup analysis showed that patients receiving surgery more than 4 days after CNB had a higher Ki67 increase compared with those treated with surgery within 2 days (*P* = 0.006). Besides, we classified STI into another 5 groups: 1-2 days (n = 55), 3 days (n = 50), 4 days (n = 63), 5 days (n = 52), and ≥ 6 days (n = 56). ANOVA analysis still showed that Ki67 change after CNB was significantly associated with STI (P = 0.01, Additional file 1: Figure S1). Luminal B-HER2- tumors, which had a higher baseline Ki67 value than Luminal A disease, showed a Ki67 decrease after CNB. However, other breast cancer subtypes showed an increased Ki67 value, with a mean Ki67 absolute increase from 3.3 % to 6.2 % (Table 5). Subgroup comparison showed that Luminal A tumors had a higher Ki67 value increase after CNB than Luminal B-HER2- (*P* = 0.014).

**Table 3:** Univariate analysis of Ki67 change and clinic-pathological factors

Clinic-pathological factors	*P* value
Age	0.521
Menopause status	0.638
Tumor size	0.658
Lymph node status	0.358
Estrogen receptor^a^	0.529
Progesterone receptor^a^	0.585
HER2^a^	0.569
Molecular subtype^a^	0.030
Surgery time interval	0.007

^a^Expression status in CNB sample

Univariate ANOVA analysis used to analyze association between Ki67 change and clinico-pathological factors

**Table 4:** Ki67 expression and change value at CNB and SRB in different surgery time intervals

	No.	Median Ki67 % (IQR)	Mean Ki67 % (SD)
All populations	276		
CNB		20 (10, 40)	26.2 (22.0)
SRS		25 (10, 40)	29.1 (22.0)
Ki67 change^a^		0 (-4.5. 10)	2.9 (13.2)
1-2 days	55		
CNB		20 (10, 40)	27.7 (21.0)
SRS		20 (10, 40)	26.6 (19.1)
Ki67 change^a^		0 (-10, 5)	-1.1 (11.0)
3-4 days	113		
CNB		20 (10, 50)	28.4 (24.6)
SRS		25 (10, 50)	30.5 (23.0)
Ki67 change^a^		0 (-5, 10)	2.1 (15.2)
≥5 days	108		
CNB		17.5 (10, 30)	23.2 (20.8)
SRS		25 (10, 40)	28.8 (22.4)
Ki67 change^a^		5 (0, 10)	5.6 (11.4)

^a^Ki67 change, SRS minus CNB

Abbreviation: *CNB* core needle biopsy, *IQR* inter quartile range, *SRS* surgically removed samples, *SD* standard deviation

**Table 5:** Ki67 expression and change value of CNB and SRS among molecular subtypes

	No.	Median Ki67 (IQR)	Mean Ki67(SD)
Luminal A	73		
CNB		10 (5, 10)	8.2 (4.1)
SRS		10 (10, 20)	14.5 (9.6)
Ki67 change^a^		5 (0, 10)	6.3 (8.6)
Luminal B HER2-	109		
CNB		20 (10, 40)	26.9 (19.5)
SRS		20 (10, 30)	26.9 (19.2)
Ki67 change^a^		0 (-10, 10)	-0.1 (12.8)
Luminal B HER2+	33		
CNB		20 (10, 50)	30.2 (20.7)
SRS		30 (15, 55)	33.5 (20.0)
Ki67 change^a^		0 (-10, 14.5)	3.3 (15.1)
Triple negative	35		
CNB		60 (30, 80)	54.1 (26.4)
SRS		70 (30, 80)	57.4 (25.8)
Ki67 change^a^		0 (-10, 10)	3.3 (13.5)
HER2 positive	26		
CNB		25 (20, 40)	31.2 (17.3)
SRS		30 (23.75, 50)	35.4 (15.1)
Ki67 change^a^		5 (-1.25, 20.0)	4.2 (19.6)

^a^Ki67 change, SRS minus CNB

Abbreviation: *CNB* core needle biopsy, *IQR* inter quartile range, *SRS* surgically removed samples, *SD* standard deviation

**Fig. 4:**
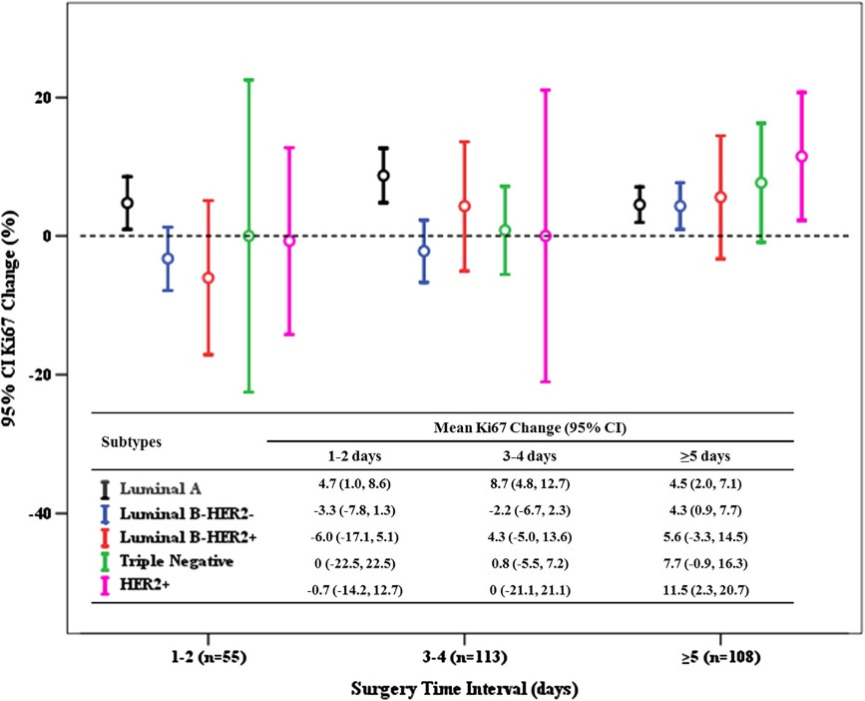
Mean Ki67 change after core needle biopsy among molecular subtypes with different surgery time intervals

Multivariate ANOVA analysis demonstrated that STI and molecular subtype were still significantly associated with Ki67 change after CNB, with *P* values of 0.010 and 0.042, respectively. Subgroup test showed that there was a slight trend of interaction between STI and molecular subtype for Ki67 change (*P* = 0.220). Furthermore, we analyzed Ki67 changes among various subtypes with different STI (Fig. 4). For HER2+ or TN patients, Ki67 change was apt to be 0 among those receiving surgery within 4 days after CNB, while the Ki67 value increase was 11.5 % and 7.7 %, respectively, in those with a STI of more than 4 days (Fig. 4).

## Discussion

Our present study demonstrated that CNB was accurate in evaluating breast cancer receptor and molecular subtype status compared with subsequent SRS. More importantly, we found that both STI and molecular subtype were associated with Ki67 changes after CNB. Luminal A tumors had more Ki67 elevation than did Luminal B-HER2- tumors. The Ki67 value increase was much more obvious in patients with prolonged STI. TN or HER2+ breast cancer patients were more likely to experience a significant Ki67 increase with a long surgery waiting time.

CNB has been proven to be a minimally invasive and accurate method in preoperative pathological diagnosis [15], and can provide sufficient tissue for breast cancer biomarkers analysis, including ER, PR, HER2, and Ki67 [1]. Meta-analysis has shown that CNB is reliable in testing ER, PR, and HER2 status compared with SRS [4]. Moreover, CNB has good agreement with SRS in breast cancer molecular subtype analysis in determining ER, PR, HER2, and Ki67 results [11]. There is no consensus concerning the Ki67 cutoff value for high proliferation [3]. In our study, we used the 20 % as the cutoff value, the median value in CNB samples and which had a relatively high concordance rate for molecular subtype analysis between CNB and SRS. The 2013 St. Gallen breast cancer consensus of 20 % Ki67 cutoff value was then applied to classify patients into various molecular subtypes. Our concordance analysis demonstrated that CNB was accurate in determining molecular subtype status compared with SRS.

Ki67, a well-established proliferation marker, is used to determine specific breast cancer Luminal subtypes [16]. For early breast cancer patients, high Ki67 expression is associated with a poor outcome [17]. Several retrospective studies have found that Ki67 could predict endocrine treatment and chemotherapy response in ER+ breast cancer patients [8, 18]. In addition, Ki67 is a key proliferation marker for calculating breast cancer recurrence score in the Oncotype-DX assay [19]. However, several studies have found that there was only fair to moderate agreement for Ki67 testing between CNB and SRS, and that the κ value was much lower than ER, PR, and HER2 evaluation; this was mainly interpreted as sampling error or tumor heterogeneity [11, 12]. Most studies have demonstrated that Ki67 expression will increase after CNB, possibly due to wound healing [20] or sample fixation intervals difference between CNB and surgically removed samples, which warrants further study. Our current data also showed a significantly higher Ki67 expression value in SRS compared with CNB samples (29.1 % vs. 26.2 %).

In preoperative “window of opportunity” clinical studies, Ki67 is often used as a surrogate biomarker to evaluate new anticancer drug anti-proliferation ability [7]. Patients treated with neoadjuvant endocrine therapy have a higher response rate if they experience a Ki67 decrease after 2 weeks therapy [8]. However, all such studies omitted Ki67 change after CNB, which may result in a shift of efficacy analysis. We analyzed potential clinic-pathological factors associated with Ki67 change after CNB to determine if any subgroup might change significantly and found that breast cancer molecular subtype was, indeed, an independent factor. Patients with different subtypes did have various Ki67 changes, indicating that Ki67 change seemed to be associated with its specific tumor biologic behavior. In neoadjuvant treatment of HER2-positive breast cancer, gene expression profiling analysis has demonstrated that subtype status can significantly change after treatment. Luminal B or HER2-enriched tumors on CNB could become Luminal A, perhaps due to cell reprogramming, stromal alteration, or heterogeneity. Luminal A tumors on CNB had more discordant cases and changes to other subtypes in SRS [21]. Our present study showed that Luminal A tumors had a higher Ki67 increase after CNB compared with Luminal B-HER2-, perhaps also reflecting wound healing, stromal reaction, or tumor heterogeneity. This indicates that further translational research needs to interpret anti-proliferation efficacy among the different molecular subtypes.

It has been reported that Ki67 changes after CNB were more obvious in TN or HER2+ breast cancer, but that there was no significant Ki67 increase in the luminal subtypes [13]. The major difference between the two studies was STI after CNB. There were 41 days in that study, much longer than in our study, which had a mean of only 4.5 days. Although, no significant Ki67 changes were noted over time after CNB in that study, we here report that STI after CNB is significantly associated with Ki67 change. Thus, breast cancer patients with longer surgery waiting time after CNB had a higher chance of Ki67 increases. Furthermore, we analyzed whether STI had different effects on Ki67 change among the breast cancer molecular subtypes. Ki67 change was apt to be 0 in TN and HER2 breast cancer patients treated with surgery within 4 days after CNB, while this was ≥ 7 % in patients with STI more than 4 days, similar to the other study. Although Ki67 increase after CNB has not been reported to cause a worse disease outcome, its increase has still been associated with chemotherapy sensitivity [10]. Moreover, a long interval between surgery and adjuvant chemotherapy has been reported to cause a worse prognosis in HER2+ and TN breast cancer patients [22]. Taken together, our data support that TN or HER2+ breast cancer patients may need to be treated with surgery within a short interval after CNB, and that neoadjuvant systemic therapy may be a reasonable option for these patients if they must wait a long time for surgery [23].

There were several limitations in our study. First, the number of enrolled patients was not large enough to further explore subgroup analysis differences, especially for subgroup interaction effect analysis. There were relatively few patients in separate subgroups regarding various STI and molecular subtype combinations. In addition, most patients were treated with surgery with a very short waiting time after CNB, which may be different from other centers. In our center, we firstly did diagnostic hematoxylin and eosin staining for tumor within 24 h after biopsy in the in-patient ward, and further surgery was arranged for most patients when invasive breast cancer was found. ER, PR, and HER2 status results was not mandatory before surgery. For other centers with longer waiting periods after biopsy, in which IHC or FISH analysis was done before surgery, one would to consider the STI differences in order to compare our results to theirs. Our mean STI was 4.5 days and only 3 separate groups were constructed with different STIs. We have not further categorized patients into another group with longer STI because there were only 10 patients with STI of more than 10 days. This prevented our study from being able to answer whether Ki67 change would be decreased in specific subtypes with a prolonged surgical waiting period. Finally, HER2 status was only evaluated by the 2007 edition of ASCO/CAP criteria, with there being a slight difference with new edition of ASCO/CAP recommendation [24]. However, we doubt that this would change our results.

## Conclusion

Our study has demonstrated that CNB is accurate in evaluating ER, PR, HER2, and molecular subtype status in breast cancer. Ki67 value can significantly increase after CNB, and this is associated with STI and molecular subtype. Luminal A tumors experienced more Ki67 elevation than did Luminal B-HER2-. Breast cancer patients with longer STI had a higher Ki67 increase after CNB. TN and HER2+ breast cancer patients with longer STI had a higher degree of Ki67 increase, while this is 0 with a short STI. We propose that anti-proliferation efficacy of anti-cancer agents needs to be evaluated among different subtypes in preoperative translational trials. Patients with TN or HER2+ breast cancer probably need to be treated with a short STI or primary systemic therapy after CNB, but this warrants further validation.

## Additional file

Additional file 1: Figure S1. Mean Ki67 change after core needle biopsy among different 5 surgery time interval groups. (TIFF 39 kb)
