# Supplementary figures and images for: Surgery time interval and molecular subtype may influence Ki67 change after core needle biopsy in breast cancer patients
Source: BMC Cancer. 2015 Oct 30;15:822. doi: 10.1186/s12885-015-1853-1 (PMC4627413; doi:10.1186/s12885-015-1853-1)

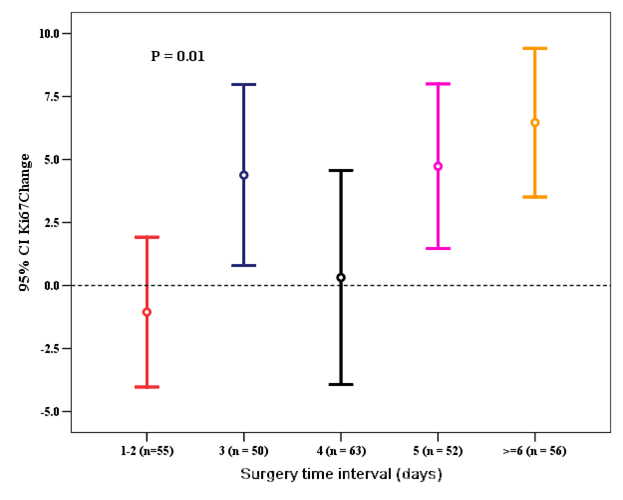

Supplement: Additional file 1: Figure S1. — Mean Ki67 change after core needle biopsy among different 5 surgery time interval groups. (TIFF 39 kb) [file 12885_2015_1853_MOESM1_ESM.tiff]
